# Integrative Analysis of *LGR5/6* Gene Variants, Gut Microbiota Composition and Osteoporosis Risk in Elderly Population

**DOI:** 10.3389/fmicb.2021.765008

**Published:** 2021-11-02

**Authors:** Dong-sheng Di, Can Li, Yu Dai, Mu-hong Wei, Shan-shan Wang, Wen-jing Song, Hao-long Zhou, Yuan Cui, Ru-yi Zhang, Qin Huang, Qi Wang

**Affiliations:** ^1^MOE Key Lab of Environment and Health, Department of Epidemiology and Biostatistics, School of Public Health, Tongji Medical College, Huazhong University of Science and Technology, Wuhan, China; ^2^Department of Cancer Prevention and Control, Hunan Cancer Hospital/The Affiliated Cancer Hospital of Xiangya School of Medicine, Central South University, Changsha, China; ^3^Department of Nuclear Medicine, Union Hospital, Tongji Medical College, Huazhong University of Science and Technology, Wuhan, China; ^4^Department of Nutrition and Food Hygiene, School of Public Health, Tongji Medical College, Huazhong University of Science and Technology, Wuhan, China; ^5^Department of Rehabilitation Medicine, Union Hospital, Tongji Medical College, Huazhong University of Science and Technology, Wuhan, China

**Keywords:** R-spondin/Wnt pathway, gut microbiota, osteoporosis, host genetics, *LGR5*, *LGR6*

## Abstract

**Objective:** This study aimed to explore the relationships between the common variants of R-spondin/Wnt signaling genes, gut microbiota composition, and osteoporosis (OP) risk in elderly Chinese Han population.

**Design:** Dual-energy X-ray absorptiometry was used to obtain the OP-associated measurements at multiple skeleton sites among all 1,168 participants. Genotyping data was obtained by using the next-generation sequencing in the discovery stage (*n* = 400, 228 OP patients) and SNPscan technology in the replication stage (*n* = 768, 356 OP patients). Bioinformatic analysis was performed to provide more evidence for the genotype-OP associations. The 16S ribosomal RNA gene high-throughput sequencing technology was adopted to explore OP-associated gut microbiota variations.

**Results:** The genetic variants of rs10920362 in the *LGR6* gene (*P*-FDR = 1.19 × 10^–6^) and rs11178860 in the *LGR5* gene (*P*-FDR = 1.51 × 10^–4^) were found to associate with OP risk significantly. Several microbial taxa were associated with the BMDs and T-scores at multiple skeleton sites. The associations between rs10920362 and BMD-associated microbiota maintained significance after adjusting confounders. The rs10920362 CT/TT genotype associated with a decreased relative abundance of *Actinobacteria* (β = −1.32, *P* < 0.001), *Bifidobacteriaceae* (β = −1.70, *P* < 0.001), and *Bifidobacterium* (β = −1.70, *P* < 0.001) compared to the CC genotype.

**Conclusion:** Our findings suggested that the variants loci of *LGR6* may be associate with OP pathogenesis via gut microbiota modifications. The relationship between host genetics and gut microbiome provides new perspectives about OP prevention and treatment.

## Introduction

Osteoporosis (OP) is a metabolic bone disease characterized by low bone mineral density (BMD) and microarchitectural deterioration of bony tissue, symptoms of which occur to one in five women over 70 years and two-thirds over 90 years (Kamiński et al., 2018). In the human body, bone metabolism is a dynamic process under strict and complex regulation affected by host genetic factors, environmental factors and lifestyle (Agas et al., 2013). A growing number of genome-wide association studies (GWASs) and SNPs studies demonstrated that genes in the R-spondin/wingless-related integration site (Wnt) signaling pathway associated with the risks of BMD reduction, OP and fracture (Baron and Gori, 2018; Correa-Rodríguez, 2018; Li et al., 2020). The leucine-rich repeat containing G protein-coupled receptor 5/6 (*LGR5/6*) are the most thoroughly studied members of the OP susceptibility genes in R-spondin/Wnt signaling pathway. *LGR5/6* function as receptors of the R-spondin family to regulate bone metabolism by potentiating Wnt/b-catenin signaling (Liu et al., 2019). *LGR6*-deficient mice showed nail and bone regeneration defect (Liu et al., 2019). In addition to genetic influences on OP, various data from human and animal studies have suggested that gut microbiota plays an indispensable role in regulating bone metabolism (Parvaneh et al., 2015; Yan et al., 2016; Schwarzer et al., 2018). Several taxa with altered abundance and specific functional pathways were found in low-BMD individuals from our previous study (Li et al., 2019). Similarly, another study showed that *Bifidobacterium longum* supplementation could increase bone formation, decrease bone resorption parameters (Parvaneh et al., 2015). Anyhow, these risk factors for OP have been thoroughly studied but are still far from being comprehensively understood.

Recently, many converging lines of evidence suggested that host genetics contribute to gut microbiota composition (Rawls et al., 2006; Parvaneh et al., 2015; Lim et al., 2017; Shukla et al., 2017; Kurilshikov et al., 2021), even through this way to affect the disease susceptibility. For instance, Rawls et al. (2006) discovered that the diversities between zebrafish and mice microbiota were caused by the differences of the underlying host genetics. Besides, one microbiome GWAS (mGWAS) study conducted by Blekhman et al. (2015) showed that the host genetic variation in immunity-related pathways was significantly associated with microbiome composition. Similarly, host SNPs were identified to have a great impact on the microbial abundance in another mGWAS (Davenport et al., 2015). It has been reported that specific host genetic variation may contribute to the development of metabolic syndrome by mediating gut microbial abundance and diversity (Lim et al., 2017). Interestingly, data from several studies suggested that R-spondin/Wnt signaling networks played a vital role in intestinal development and maintenance of intestinal homeostasis, as well as the development and differentiation of immune cells (Staal et al., 2008; Clevers and Nusse, 2012; Hayase et al., 2017). And beyond that, several functional studies have reported the importance of Wnt signaling in the gut microbiota–bone axis (Tyagi et al., 2018; Chen et al., 2020). Significantly, *LGR5/6* can promote R-spondin-mediated Wnt/PCP and Wnt/β-catenin signaling (Liu et al., 2019). Considering that both host genes and the microbiome influence the development of the OP, we hypothesized that host genetic variants in R-spondin/Wnt signaling pathway, especially the *LGR5/6* genes, might partly regulate bone metabolism by mediating some microbe abundance. However, no information has yet been available on the interrelationships among host genetics, gut microbiota, and OP risk. Thus, what becomes particularly important is the identification of associations between specific genetic variants and microbes involving this process.

Herein, to some degree, the present study attempted to fill the gaps of this knowledge that R-spondin/Wnt signaling networks genetic variants contribute to OP risk via controlling gut microbiota composition. Therefore, we adopted the next-generation sequencing (NGS), SNPscan and 16S ribosomal RNA (rRNA) gene high-throughput sequencing technology to conduct an integrative analysis of *LGR5/6* gene variants, gut microbiota composition, and OP risk based on a Han population of central China.

## Materials and Methods

### Study Design

**FIGURE 1:**
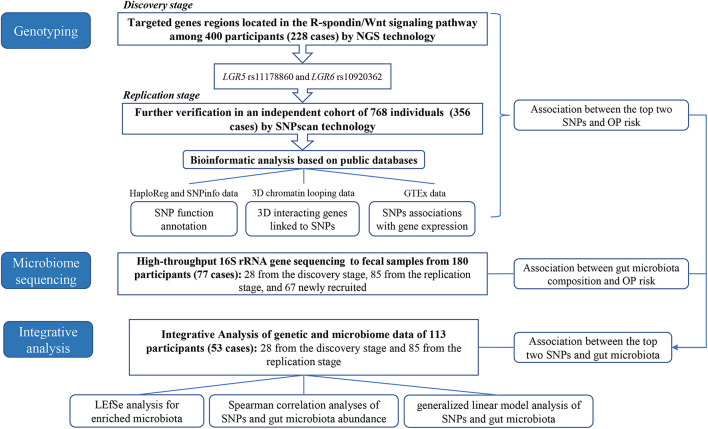
Framework of the design and data analysis of this study.

The detailed analyses process was shown in Figure 1. In the present research, we first performed the targeted NGS to the R-spondin/Wnt pathway genes in 400 Chinese participants (228 OP patients) and replicated the findings using SNPsacn technology among another independent cohort of 768 individuals (356 OP patients) in the same area. Bioinformatic analysis was then performed referring to the HaploReg, SNPinfo, 3DSNP, and Genotype-Tissue Expression (GTEx) data to provide more evidence for the OP-genotype associations. Third, the 16S rRNA gene high-throughput sequencing technology was adopted to explore OP-associated gut microbiota variations. Finally, integrative analysis was conducted combining the genotyping and microbiome data to clarify potential genome-microbiome associations in OP pathogenesis.

### Participants and Baseline Data Collection

The study involved a total of 1,168 Han Chinese participants aged ≥60 years recruited at two communities in Wuhan city and the Wuhan Union Hospital during 2016–2018. The exclusion criteria were as follows: (1) with other endocrine diseases (e.g., hyperthyroidism, hypothyroidism, etc.) that influences bone metabolism; (2) of surgical menopause (i.e., hysterectomy and/or ovariectomy); and (3) taking medicines affecting bone health such as hormones. Moreover, individuals were further excluded before stool collection if they met the following criterion: (1) use of antibiotics within 1 month before fecal sample collection; and (2) with prevalent diseases of diabetes and gastrointestinal diseases.

The covariates have been described in our previous study (Li et al., 2020). Briefly, body mass index (BMI) and data of self-reported menstrual history, lifestyle factors, prevalent diseases, and medication history were collected. This study was approved by the Ethics Committee of Tongji Medical College of Huazhong University of Science and Technology. Informed consent was obtained from all participants before enrollment.

### Bone Mineral Density Measurement

Dual-energy X-ray absorptiometry (Lunar Prodigy, GE, United States) was used to obtain BMD measurements, including values of BMD (expressed in g/cm^2^), *T*-score, and *Z*-score, at skeleton sites of the lumbar spine (LS) and total hip among all participants. A *T*-score ≤ −2.5 indicated prevalent osteoporosis referring to the World Health Organization criteria (Chen et al., 2020). Subjects with *T*-score ≤ −2.5 were grouped into OP patients group, and those with *T*-score > -2.5 were classified into the controls.

### Genotype Data

Details of venous blood sample collection, genome DNA extraction and gene sequencing methods as NGS and SNPscan were available in one of our studies (Li et al., 2020). Briefly speaking, the targeted NGS was used in the discovery stage (*N* = 400 and 228 OP patients) to reveal the OP-associated genetic variants located in the target regions of R-spondin/Wnt pathway genes including *RSPO1*, *RSPO2*, *RSPO3*, *RSPO4*, *LRP5*, *LRP6*, *LGR4*, *LGR5*, and *LGR6*. Then in the replication stage, the SNPscan^TM^ Kit (Genesky Biotechnologies Inc., Suzhou, China) was used for sequencing of the observed genetic variants in association with OP risk in an independent cohort (*N* = 768 and 356 OP patients). Since limited numbers of sample sizes in our study, we only involved common genetic variants (Minor Allele Frequency > 0.05) for analysis in the present study.

### Bioinformatic Analysis of Single Nucleotide Polymorphisms Function

The identified OP-associated SNPs and their LD proxies (*r*^2^ ≥ 0.8) were annotated for potential regulatory function by HaploReg v4.1 (Ward and Kellis, 2016). The FuncPred tool was used for functional SNPs prediction in the SNPinfo web server (Xu and Taylor, 2009). Three-dimensional (3D) chromatin looping data (Lu et al., 2017) were used to link promising SNPs to their three-dimensional interacting genes. Expression quantitative trait loci (eQTL) and splicing quantitative trait loci (sQTL) analysis were conducted on the SNPs using the GTEx project (Carithers and Moore, 2015).

### Intestinal Flora Data

The high-throughput 16S rRNA gene sequencing method was used to detect the microbiota of fecal samples collected from eligible participants in this study. Details of fecal sample collection, DNA extraction, and PCR amplification were described in one previous study (Li et al., 2019). Briefly, fecal samples were collected and stored at −80°C until further processing. The fecal microbial DNA was extracted using the QIAamp DNA Stool Mini Kit (Qiagen, Hilden, Germany). PCR amplification was carried out to the V3–V4 hypervariable region of the 16S rRNA gene with bacterial genomic DNA as template. Then, the PCR products were pooled and purified with Agencourt AMPure XP magnetic beads (Beckman Coulter, United States) using the TopTaq DNA Polymerase kit (Transgen, China). PCR products were sequenced using the Illumina Miseq platform with the 2 × 250 bp paired-end method after the library was quantified, mixed, and quality checked. To obtain clean reads, we firstly used TrimGalore^1^ to filter raw reads at Q20 and adapter sequence and removed the reads with length <100 bp. Then, FLASH2 (Magoč and Salzberg, 2011) was used to merge pairs of reads from the original DNA fragments. We further removed the low-quality sequences after merging. After that, Mothur (Schloss et al., 2009) was used to remove primers in sequence, and sequences including N-base/homopolymer > 6 bp. Reads with an error rate of >2 and reads with length <100 bp were removed using USEARCH^2^ to obtain clean reads for further analyses. Operational taxonomic units (OTUs) were assigned by clustering the sequences with a threshold of 97% pairwise identity and chimeras were removed using UPARSE (Edgar, 2013). OTUs were taxonomically assigned at a confidence threshold of 80% based on the Ribosomal Database Project database by Mothur.

### Statistical Analyses

The Pearson χ^2^ test was used to analyze the between-group differences of categorical variables (e.g., sex, smoking, and drinking), which were expressed as percentages. Two independent-sample *t*-test was used to analyze the between-group differences of variables of a normal distribution, which were expressed as mean ± standard deviation (SD). Mann–Whitney *U* test was used to analyze the between-group differences of continuous variables not conforming to a normal distribution, which were presented as median [interquartile range (IQR)]. To assess the beta diversity, a principal coordinate analysis (PCoA) based on the weighted UniFrac was performed for the visualization of the microbiome structure separation across groups. Statistical significance was confirmed using Permutational MANOVA (PERMANOVA). The linear discriminant analysis (LDA) effect size (LEfSe) was performed to identify the species accounting for the differences between groups, where the species with LDA values > 2.5 at a *P*-value < 0.05 were considered significantly enriched. Logistic regression modeling was performed to assess the associations of sequenced genetic variants with OP risk. Spearman’s correlation analysis was used to investigate the correlation between SNPs and gut microbiota. A generalized linear model based on negative binomial distribution was conducted to explore the relationship between SNPs and gut microbiota by taking the attributes of the microbiome (like the relative abundance of the microbiome) as the dependent variables, the genotypes in the host loci dominant model as the independent variables, and the gender, age, BMI, smoking, and drinking as covariates. The R software and SPSS version 23.0 were used for statistical analyses. A two-sided *P* < 0.05 was considered to achieve statistical significance.

## Results

### General Characteristics of the Subjects

The demographic characteristics of all participants were presented in Table 1. In the discovery and replication stages, the mean age of individuals in the cases with prevalent osteoporosis (OP) was slightly younger than the controls with normal BMD values. Taking the idea that women have high risk of OP prevalence into account, we involved all 400 females (228 cases and 172 controls) in the discovery stage of genotyping. And in the replication stage among 768 individuals, we also included males. It seems that the OP patients were likely to have larger BMI compared to controls. For the women studied, the cases and controls had similar mean menarche age (about 14 years old), as well as menopause age (about 49 years old). Generally speaking, the cases had higher proportions of smoking, and alcohol drinking, while lower proportions of self-reporting history of fracture and osteoarthritis compared to the controls though the differences achieved significance or not.

**TABLE 1:** Characteristics of included subjects for genotyping in the study.

**Variables**	***n***	**Discovery stage**		***P*-value**	**Replication stage**		***P*-value**
		**Controls (*n* = 172)**	**Cases (*n* = 228)**		**Controls (*n* = 412)**	**Cases (*n* = 356)**	
Age (years), mean ± *SD*	1168	66.4 ± 4.75	67.4 ± 5.76	0.142	66.7 ± 6.94	67.9 ± 6.14	0.005
Female, *n* (%)	1168	172 (100.0)	228 (100.0)	NA	148 (35.9)	219 (61.5)	1.43 × 10^–12^
BMI (kg/m^2^), mean ± *SD*	1168	25.4 ± 3.1	23.2 ± 3.1	3.89 × 10^–11^	24.9 ± 3.1	23.3 ± 3.2	1.32 × 10^–12^
Age of menopause (years), mean ± *SD*^#^	746	49.8 ± 4.38	48.9 ± 3.86	0.001	49.8 ± 3.68	49.2 ± 4.02	0.224
Age of menarche (years), mean ± *SD*^#^	733	14.1 ± 1.92	14.1 ± 1.85	0.788	14.1 ± 2.10	13.9 ± 1.78	0.851
Parity^#^	680	1.59 ± 0.80	1.72 ± 1.03	0.424	1.40 ± 0.96	1.59 ± 1.00	0.017
Smoking, *n* (%)	1146	2 (1.2)	8 (3.5)	0.246	76 (19.0)	55 (15.7)	0.224
Alcohol drinking, *n* (%)	1146	0 (0)	5 (2.2)	0.134	87 (21.8)	49 (14.0)	0.005
Fracture (%)	1159	42 (24.9)	77 (33.8)	0.055	86 (21.1)	128 (36.2)	4.00 × 10^–6^
Osteoarthritis (%)	1080	51 (30.5)	49 (22.2)	0.062	65 (17.4)	82 (25.7)	0.008

*^#^Only for women. BMI, body mass index; SD, standard deviation; NA, not applicable.*

### Associations Between Two Common Variants in *LGR5/6* and Osteoporosis Risk

Four genetic models (i.e., the additive, dominant, co-dominant, and recessive models) were employed to analyze the association between the studied SNPs and OP risk, results of which were presented in Table 2. Combing the data from both stages, it was observed in the additive model that the rs10920362 T allele carriers had a significant increased risk of OP prevalence compared to the C allele carriers (OR = 1.78, 95% CI: 1.44–2.22, *P*-FDR = 1.19 × 10^–6^). The dominant model revealed that the CT/TT genotypes were associated with larger OP risk than the CC genotypes (OR = 1.86, 95% CI: 1.44–2.41, *P*-FDR = 2.50 × 10^–5^). And compared to the CT/CC genotypes, the TT genotype was significantly associated with an increased risk of OP prevalence (OR = 3.06, 95% CI: 1.71–5.49, *P*-FDR = 8.43 × 10^–4^). As to the rs11178860, an increased risk of OP was observed in individuals with the A allele compared with the individuals carrying the G allele (OR = 1.48, 95% CI: 1.23–1.78, *P*-FDR = 1.51 × 10^–4^). Importantly, the dominant and recessive models also showed results achieve significance. Moreover, the two common SNPs were also associated with the values of BMD measurements at skeleton sites of LS and total hip (Supplementary Table 1).

**TABLE 2:** Associations between the top two common variants and osteoporosis risk.

**Genotype**	**Discovery stage [*n* (%)]**		**OR (95%CI)**	**Replication stage [*n* (%)]**		**OR (95%CI)**	**Total [*n* (%)]**		**OR (95%CI)**	***P*-value**	***P*-FDR**
	**Cases**	**Controls**		**Cases**	**Controls**		**Cases**	**Controls**			
rs10920362											
CC	112 (50.22)	99 (59.28)	1.00	178 (50.00)	278 (67.48)	1.00	290 (50.09)	376 (65.28)	1.00		
CT	92 (41.26)	64 (38.32)	1.73 (1.05, 2.87)	149 (41.58)	118 (28.64)	1.84 (1.32, 2.57)	241 (41.62)	180 (31.25)	1.80 (1.37, 2.37)	1.74 × 10^–4^	
TT	19 (8.52)	4 (2.40)	6.04 (1.82, 20.1)	29 (8.15)	16 (3.88)	3.36 (1.67, 6.73)	48 (8.29)	20 (3.47)	3.02 (1.71, 5.32)	1.40 × 10^–5^	
Additive model			2.01 (1.33, 3.02)			1.86 (1.43, 2.42)			1.78 (1.44, 2.22)	1.19 × 10^–7^	1.19 × 10^–6^
Dominant model			2.01 (1.23, 3.29)			2.04 (1.48, 2.81)			1.86 (1.44, 2.41)	2.50 × 10^–6^	2.50 × 10^–5^
Recessive model			4.80 (1.48, 15.6)			2.66 (1.34, 5.27)			3.06 (1.71, 5.49)	1.69 × 10^–4^	8.43 × 10^–4^
rs11178860											
GG	67 (30.18)	58 (34.12)	1.00	87 (24.44)	154 (37.78)	1.00	154 (26.64)	212 (36.43)	1.00		
GA	98 (44.14)	86 (50.59)	0.84 (0.49, 1.44)	192 (53.93)	206 (50.00)	1.56 (1.09, 2.23)	290 (50.17)	292 (50.17)	1.29 (0.97, 1.72)	0.086	
AA	57 (25.68)	26 (15.29)	1.82 (0.93, 3.55)	77 (21.63)	52 (12.62)	2.53 (1.56, 4.08)	134 (23.18)	78 (13.40)	2.30 (1.58, 3.36)	1.50 × 10^–5^	
Additive model			1.32 (0.95, 1.83)			1.59 (1.26, 2.00)			1.48 (1.23, 1.78)	3.02 × 10^–5^	1.51 × 10^–4^
Dominant model			1.12 (0.68, 1.86)			1.75 (1.25, 2.46)			1.50 (1.14, 1.97)	3.49 × 10^–3^	0.017
Recessive model			2.07 (1.14, 3.76)			1.91 (1.25, 2.92)			1.97 (1.41, 2.75)	6.95 × 10^–5^	6.95 × 10^–4^

*SNP, single nucleotide polymorphisms; *P*-FDR were Benjamini–Hochberg false discovery rate (FDR)-corrected. Covariates including sex, age, BMI, smoking and drinking were adjusted.*

### Annotation and Function Assessment of the Two Osteoporosis-Associated Genetic Variants

Referring to the predictions by HaploReg v4.1, the variant rs11178860 was a proxy (*r*^2^ > 0.8) of genetic variants locating in a variety of regulatory elements of “Promoter histone marks,” “Enhancer histone marks,” “Motifs changed,” “GRASP QTL hits,” and “Selected eQTL hits” (Supplementary Table 2). Besides, the SNP rs10920362 was predicted to function in regulatory elements of “DNAse,” “Enhancer histone mark,” “Proteins bound,” “Motifs changed,” “GRASP QTL hits,” and “Selected eQTL hits” (Supplementary Table 2). According to the functional analysis by SNPinfo, the rs10920362 was identified as “possibly damaging” with features of an exonic splicing enhancer or exonic splicing silencer (Supplementary Table 3). Transcription factor binding sites (TFBSs) were identified for the rs2304269 of strong LD with the rs11178860 (*r*^2^ = 0.814 in CHB) (Supplementary Table 3). The 3D chromatin looping data in bone demonstrated that the interacting genes of rs10920362 are *LGR6* and *SYT2*, and rs10920362 was linked with 14 TFBSs (Supplementary Figure 1).

Based on the GTEx datasets, we analyzed the correlation between the two genetic variants (rs10920362 and rs11178860) and gene expressions in various tissues, results of which are shown in Supplementary Table 4. We observed potential associations between rs10920362 and the *LGR6* gene expression in tissues of thyroid (*P* = 7.20 × 10^–39^), brain hypothalamus (*P* = 1.10 × 10^–11^), minor salivary gland (*P* = 2.10 × 10^–9^), artery tibial (*P* = 1.00 × 10^–8^), brain substantia nigra (*P* = 3.00 × 10^–7^), artery aorta (*P* = 4.20 × 10^–7^), and brain cortex (*P* = 4.30 × 10^–5^). We failed to find the rs11178860 and *LGR5* gene expression association, but the sQTL data showed a potential association between rs10879301 (of large LD with rs11178860: *r*^2^ = 1.00 in CHB) and the splicing changes of the *LGR5* gene in the muscle-skeletal tissue (Supplementary Figure 2).

### Osteoporosis Risk-Associated Variations in Gut Microbiota

A total of 180 fecal samples from 77 OP patients and 103 controls were included in the gut microbiota abundance analysis (Supplementary Table 5). The OP group had comparatively smaller numbers of bacterial taxa at species, genus, family, order, class, and phylum levels than the control group (Supplementary Table 6). A total of 1,699 OTUs were quantified in this study, specifically, 1,288 in the OP group and 1,556 in the control group with 1,145 shared in both groups.

The PCoA of weighted UniFrac distance showed a significant difference between the OP and control groups (Supplementary Figure 3). The PC1 explained 46.64% of variation and the PC2 explained 10.53%. The PERMANOVA analysis also revealed that the two groups had a significant difference in beta diversity (*F* = 3.413, *P* = 1.0 × 10^–4^). LEfSe analysis revealed that the Bacteroidetes (phylum), Porphyromonadaceae (family), Bacteroidaceae (family), *Parabacteroides* (genus), and *Bacteroides* (genus) were enriched in the OP individuals; and the Firmicutes (phylum), Proteobacteria (phylum), Actinobacteria (phylum), Lachnospiraceae (family), Bifidobacteriaceae (family), *Butyricicoccus* (genus) and *Bifidobacterium* (genus) were enriched in the controls (Supplementary Figure 4). The Firmicutes, Bacteroidetes, Proteobacteria, and Actinobacteria mainly dominated at phylum level in gut microbiota of all samples. The proportion of Bacteroidetes was significantly larger in the OP group than that in the control group (*P* < 0.05), while those of the Firmicutes, Proteobacteria, and Actinobacteria were smaller in the former than the latter (all *P* < 0.05) (Table 3). At family level, the OP patients had a higher proportion of Bacteroidaceae and Porphyromonadaceae than the controls (both *P* < 0.05). At genus level, the *Bacteroides* and *Parabacteroides* accounted for a larger proportion in the case group than the controls (both *P* < 0.05). Spearman’s correlation analysis observed that levels of the Firmicutes, Proteobacteria, and Actinobacteria phylum, the Bifidobacteriaceae, Lactobacillaceae, Ruminococcaceae, and Ruminococcaceae families, and the *Bifidobacterium*, *Lactobacillus*, and *Gemmiger* genus correlated positively with the BMDs and *T*-scores among all subjects (Supplementary Table 7). Of note, the ridge regression model still showed significant associations between gut microbiota and skeleton site-specific BMD measurements after adjusting for age, sex, smoking, and BMI (Supplementary Table 8).

**TABLE 3:** Comparisons of gut microbiota composition at several taxonomic levels between the osteoporosis patients (cases) and controls.

**Taxonomic level**	**Median (IQR)**		***P*-value**
	**Cases**	**Controls**	
**Phylum**			
Firmicutes	16097 (16336)	21818 (16413)	0.001
Bacteroidetes	21947 (17574)	11503 (21249)	1.96 × 10^–6^
Proteobacteria	922 (1623)	1660 (3134)	0.004
Actinobacteria	163 (850)	570 (2508)	0.007
**Family**			
Bacteroidaceae	15449 (13683)	7054 (13957)	1.65 × 10^–6^
Lachnospiraceae	6231 (9896)	8597 (9835)	0.004
Enterobacteriaceae	230 (1072)	700 (2737)	0.009
Bifidobacteriaceae	57 (444)	254 (1564)	0.003
Porphyromonadaceae	1294 (1830)	607 (1695)	0.008
Lactobacillaceae	2 (9)	5 (51)	0.011
**Genus**			
*Bacteroides*	15449 (29)	7054 (13957)	1.65 × 10^–6^
*Blautia*	314 (2508)	1321 (4379)	0.026
*Bifidobacterium*	57 (443)	254 (1564)	0.003
*Parabacteroides*	751 (1196)	432 (809)	0.003
*Lactobacillus*	2 (9)	5 (51)	0.011

*IQR, interquartile range.*

*Only the phyla, family, and genera with relative abundance greater than 0.1% were included in this analysis.*

### Integrative Analysis of Osteoporosis-Associated Genetic Variants and Gut Microbiota

**FIGURE 2:**
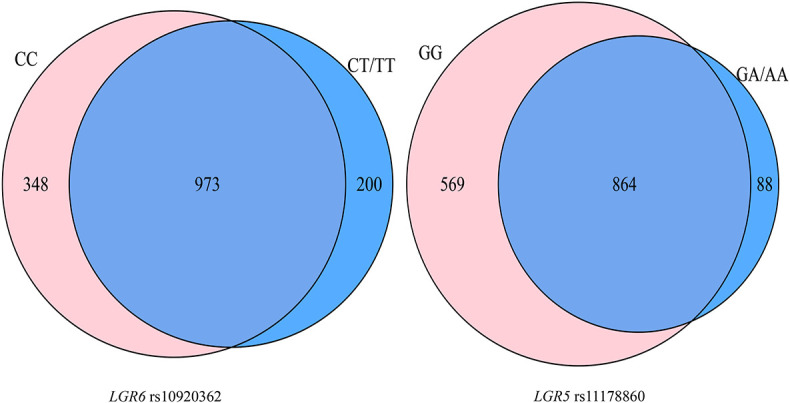
Gut microbiota diversity in the SNPs genotype at the operational taxonomic units level.

A total of 113 participants (53 OP patients) simultaneously with genetic and gut microbiota data were included in this part. A total of 1,521 OTUs were identified. The Venn diagram showed that the rs10920362 CC and CT/TT carriers shared 973 (64.0%) OTUs with 348 found only in the CC carriers and 200 found only in the CT/TT carriers (Figure 2). The CC carriers had more OTUs (1,321) than the CT/TT carriers (1,173). A total of 864 (56.8%) OTUs were shared across the rs11178860 GG and GA/AA genotypes with 569 OTUs only found in the GG genotype individuals and 88 found only in the GA/AA genotype individuals. The number of OTUs was larger in the GG genotypes (1,433) than the GA/AA genotypes (952).

**FIGURE 3:**
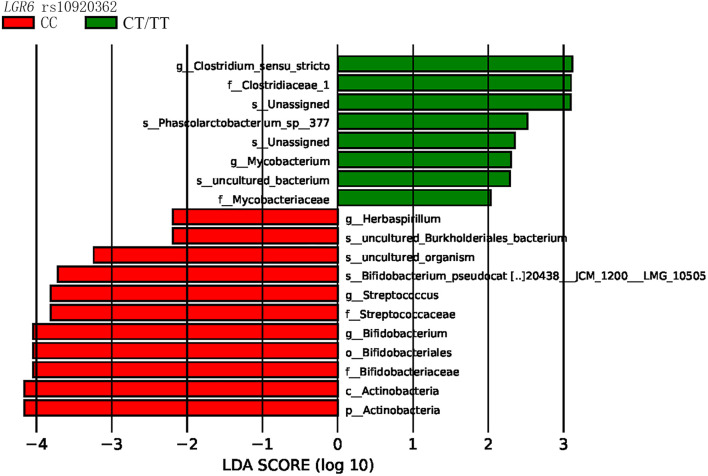
LEfSe indicating differences in the bacterial taxa in different rs10920362 genotypes (p, phylum; c, class; o, order; f, family; g, genus; and s, species), only the taxes having a *P* < 0.05, and LDA value > 2 are shown in the figure.

LEfSe analysis revealed that the bacterial species belonging to phylum Actinobacteria, family Bifidobacteriaceae, and family Streptococcaceae were enriched in the *LGR6* rs10920362 CC genotype. Compared to the *LGR6* rs10920362 CT/TT genotype individuals, CC genotype individuals had a higher level of *Bifidobacterium* and *Streptococcus* (Figure 3). As for rs11178860, no significant differences were found.

As data shown in further analyses, the proportion of Actinobacteria in the rs10920362 CT/TT genotype was significantly lower than that in the CC genotype at the phylum level (*P* = 0.005) (Table 4). At the family level, the individuals carrying CT/TT genotype had lower abundances of Bifidobacteriaceae compared to those with CC genotype (*P* = 0.002). At the genus level, individuals with CT/TT genotype had lower abundances of *Bifidobacterium* than individuals with CC genotype (*P* = 0.002). However, no significant differences in gut microbial community abundance were found between rs11178860 GG and GA/AA genotypes. Of note, the abundances of Actinobacteria, Bifidobacteriaceae, and *Bifidobacterium* were also positively correlated with BMD and T-score (Li et al., 2019).

**TABLE 4:** Comparisons of gut microbiota composition stratified by the rs10920362 and rs11178860 genotypes.

	**rs10920362 [median (IQR)]**			**rs11178860 [median (IQR)]**		
	**CC**	**CT/TT**	***P*-value**	**GG**	**GA/AA**	***P*-value**
p-Firmicutes	16688 (16583)	15014 (11861)	0.208	13893 (16301)	16097 (15624)	0.602
p-Bacteroidetes	21947 (20873)	25124 (13545)	0.181	23998 (16374)	22942 (18077)	0.528
p-Proteobacteria	1380 (2615)	1486 (1696)	0.943	1259 (2415)	1390 (2213)	0.868
p-Actinobacteria	229 (1091)	88 (301)	**0.005**	170 (885)	146 (678)	0.480
f-Bacteroidaceae	14207 (18658)	17967 (12936)	0.298	15044 (13421)	16423 (18658)	0.913
f-Lachnospiraceae	6386 (7468)	5397 (8422)	0.436	5872 (6437)	6288 (9278)	0.46
f-Ruminococcaceae	4702 (5494)	4500 (8151)	0.785	3721 (5881)	4871 (5201)	0.637
f-Enterobacteriaceae	366 (1984)	220 (1527)	0.363	391 (1963)	296 (1517)	0.971
f-Bifidobacteriaceae	110 (901)	32 (203)	**0.002**	63 (785)	73 (511)	0.554
f-Lactobacillaceae	2 (8)	2 (20)	0.582	3 (18)	2 (9)	0.647
g-Bacteroides	14207 (18658)	17967 (12936)	0.298	15044 (13421)	16423 (18658)	0.913
g-Blautia	267 (1942)	239 (1563)	0.454	292 (1452)	267 (2115)	0.301
g-Prevotella	9 (506)	7 (2609)	0.889	40 (1401)	7 (1115)	0.181
g-Bifidobacterium	110 (901)	32 (203)	**0.002**	63 (785)	73 (511)	0.557
g-Parabacteroides	734 (1354)	894 (1700)	0.726	848 (1409)	735 (1398)	0.555
g-Ruminococcus	242 (1009)	529 (1441)	0.067	377 (1167)	411 (1148)	0.939
g-Lactobacillus	2 (8)	2 (20)	0.582	3 (18)	2 (9)	0.647
g-Roseburia	136 (521)	166 (559)	0.605	156 (470)	155 (582)	0.477
g-Dialister	33 (806)	47 (507)	0.582	45 (914)	34 (674)	0.530

*Data not following a normal distribution were expressed by median (IQR), and analyzed by using the Mann–Whitney *U* test.*

*Only the phyla, family, and genera with relative abundance greater than 1% were included in this analysis.*

*Bold values indicated a statistical significance of *P* < 0.05.*

*IQR, interquartile range; p, phylum; f, family; g, genus.*

Spearman’s correlation analysis showed that the abundances of Actinobacteria, Bifidobacteriaceae, and *Bifidobacterium* correlated negatively with the rs10920362 CT/TT genotype (all *P* < 0.05) (Table 5). No significant correlation was found between SNP rs11178860 and gut microbiota abundance.

**TABLE 5:** Spearman’s correlation analysis of the two common variants and gut microbiota abundance.

**Taxonomic level**	**rs10920362**		**rs11178860**	
	**ρ**	***P-*value**	**ρ**	***P-*value**
p-Firmicutes	–0.119	0.209	0.049	0.604
p-Bacteroidetes	0.126	0.182	–0.060	0.531
p-Proteobacteria	0.007	0.944	0.016	0.868
p-Actinobacteria	–0.264	**0.005**	0.067	0.482
f-Bacteroidaceae	0.098	0.301	–0.010	0.914
f-Lachnospiraceae	–0.074	0.439	0.070	0.462
f-Ruminococcaceae	–0.026	0.786	0.045	0.640
f-Enterobacteriaceae	–0.086	0.366	0.003	0.971
f-Bifidobacteriaceae	–0.290	**0.002**	0.056	0.557
f-Lactobacillaceae	0.052	0.584	–0.043	0.649
g-Bacteroides	0.098	0.301	–0.010	0.914
g-Blautia	–0.071	0.457	0.098	0.304
g-Prevotella	0.013	0.890	–0.126	0.183
g-Bifidobacterium	–0.290	**0.002**	0.056	0.559
g-Parabacteroides	0.033	0.728	–0.056	0.557
g-Ruminococcus	0.173	0.067	0.007	0.940
g-Lactobacillus	0.052	0.584	–0.043	0.649
g-Roseburia	0.049	0.607	0.067	0.480
g-Dialister	–0.052	0.584	–0.059	0.532

*Only the phyla, family, and genera with relative abundance greater than 1% were included in this analysis.*

*Bold values indicated a statistically significant of *P* < 0.05.*

*ρ, Spearman’s rank correlation coefficient; p, phylum; f, family; g, genus.*

Based on above results, we took the relative abundance of Actinobacteria, Bifidobacteriaceae, and *Bifidobacterium* as dependent variables, and the rs10920362 and rs11178860 genotypes as independent variables in the generalized linear modeling. Compared with the rs10920362 CC genotype, the CT/TT genotype associated with decreased relative abundance of Actinobacteria (β = −1.32, *P* < 0.001), Bifidobacteriaceae (β = −1.70, *P* < 0.001), and *Bifidobacterium* (β = −1.70, *P* < 0.001) controlling for sex, age, BMI, cigarette smoking, alcohol drinking, and self-reporting history of osteoarthritis (Table 6). No significant associations were found between the rs11178860 genotype and the relative abundance of above-mentioned gut microbiota.

**TABLE 6:** Results of generalized linear model analysis of the dependency of gut microbiota abundance on the two genetic variants*.

**SNPs**	**Genotype**	**Actinobacteria**		**Bifidobacteriaceae**		***Bifidobacterium***	
		**β (95% CI)**	***P*-value**	**β (95% CI)**	***P*-value**	**β (95% CI)**	***P*-value**
rs10920362	CC						
	CT/TT	−1.32 (−1.80, −0.84)	<0.001	−1.70 (−2.19, −1.21)	<0.001	−1.70 (−2.19, −1.21)	<0.001
rs11178860	GG						
	GA/AA	0.49 (−0.31, 1.29)	0.229	0.08 (−0.72, 0.88)	0.844	0.09 (−0.71, 0.89)	0.829

*Generalized linear model based on negative binomial distribution was performed adjusting for sex, age, BMI, cigarette smoking, alcohol drinking, and self-reporting history of osteoarthritis.

*SNP, single nucleotide polymorphisms; β, regression coefficients; CI, confidence interval.*

## Discussion

In this study, we conducted an integrative analysis of R-spondin/Wnt signaling network gene polymorphisms and gut microbiota composition in relation to the OP risk among a Han Chinese population in central China. As we knew, it was firstly reported that the genetic variants of rs11178860 in *LGR5* gene and rs10920362 in *LGR6* gene associated with OP prevalence risk among Chinese elderly individuals. Besides, the abundance of several bacterial taxa was revealed to be associated with OP prevalence risk and/or relevant disease traits as BMDs and *T*-scores at the studied skeleton sites. Importantly, we showed for the first time that the rs10920362 polymorphism was significantly associated with several BMD-associated microbial taxa including Actinobacteria, Bifidobacteriaceae, and *Bifidobacterium*. In short, we provided epidemiological evidence that two genetic variants in two genes of R-spondin/Wnt signaling networks contribute to OP risk possibly via biological influence on gut microbiota compositions.

The associations between the two common SNPs and OP risk were genuinely supported by evidence from previous functional studies (Cui et al., 2018; Lin et al., 2019) and bioinformatics analysis in our study. Specifically, the Haploreg predicted that mutations in the two gene loci (or those in high LD with them) can cause disrupted transcription factor binding sites (Ward and Kellis, 2016). That was also evidenced by predictions of the SNPinfo (Xu and Taylor, 2009) and 3DSNP (Lu et al., 2017) web servers. Consistently, the GTEx data suggested that rs10920362 act as an eQTL for the *LGR6* gene across various tissues. Besides, the rs11178860 was a proxy of a genetic variant (the rs10879301 in LD with rs11178860) predicted to associate with the splicing changes of *LGR5* gene in the muscle-skeletal tissue. Other functional studies also provided consistent findings. It was demonstrated that inhibition of *LGR6* promoted the osteogenic differentiation of bone marrow stromal cells *in vitro* (Cui et al., 2018). Moreover, transplantation of *LGR6*-knockout bone marrow stromal cells in rat models contributed to better recovery after the fracture (Cui et al., 2018). Lin et al. (2019) showed that Lgr5 was implicated in the cellular processes of osteogenic differentiation of mesenchymal stem cells by regulating Wnt and ERK signaling pathways and mitochondrial dynamics in fusion and fission. Inhibition of Lgr5 expression induced elevations in mitochondrial fragmentation and suppression of osteogenesis (Lin et al., 2019). Taken together, the functional links of rs10920362 and rs11178860 with OP pathogenesis are biologically convincing. Furthermore, the consistent results of *LGR6* rs10920362 and *LGR5* rs11178860 in the discovery and replication stages supported the credibility of the findings. Genotype-phenotype associations between risk alleles and disease subtypes may provide insight into disease etiology and mechanisms. Intriguingly, the significant associations between the two common variants and BMDs at diverse skeleton sites suggested that our findings are tenable from other perspectives. Overall, multiple lines of evidence strengthened the effectiveness and credibility of the association between *LGR6* rs10920362 and *LGR5* rs11178860 and OP risk.

Our results showed that the rs10920362 polymorphism were also associated with the abundance of several BMD-associated microbial taxa like the Actinobacteria, Bifidobacteriaceae, and *Bifidobacterium*. Genetic variants associated with the quantitative traits of microbiome are defined as microbiome QTLs (mQTLs) (Bloomgarden, 2018; Cavadas et al., 2020). Several studies also reported the role of genetic variants affecting gut microbiota composition (Benson et al., 2010; McKnite et al., 2012; Goodrich et al., 2014). Animal studies revealed numerous loci associated with gut microbial community composition by using QTL-mapping approaches, some of which overlapped genes involved in immune response (Benson et al., 2010; McKnite et al., 2012). A metagenomic sequencing research to 1,514 individuals also evidenced associations between genetic variants and gut microbiome composition and function, indicating potential interactions between host genome and microbiome in human (Bonder et al., 2016). Several studies have identified mQTLs in relation to human diseases, including IBD, cancer, heart disease, and meningitis (Hillhouse et al., 2011; Snijders et al., 2016). These mQTLs can be regulated by genes involved in microbiome-related pathways, including the immune system, food metabolism, and drug-related systems. Interestingly, a functional study of Chen et al. (2020) found that the *Clostridium butyricum*, a butyrate-producing probiotics, can modulate Wnt signaling and gut microbiota. Similarly, another study of Tyagi et al. (2018) showed that the butyrate indirectly increased CD8^+^ T cell expression of Wnt10b via T_reg_ cells, suggesting the importance of Wnt signaling in the gut microbiota–bone axis. Furthermore, *LGR6* functioned as a receptor of RSPOs to potentiate Wnt/β-catenin signaling (Gong et al., 2012). Therefore, we believe it is reasonable to consider that the *LGR6* gene is capable of regulating gut microbiota composition through some underlying mechanisms possibly relevant to particular mQTLs. However, regarding the identified association between rs10920362 and OP-associated gut microbiota, functional studies are still required to confirm a QTL effect of the *LGR6* rs10920362 mutation and elucidate the molecular mechanism through which this QTL acts.

So far, the underlying mechanisms of gut microbiota variations in association with BMD decline still remain unclear. One reasonable explanation is that the overproduction of lipopolysaccharide from the gut microbiota possibly contributes to bone mass loss via inflammation-relevant pathways (Itoh et al., 2003). What is more, the high heritability of *Bifidobacterium*, Bifidobacteriaceae, and Actinobacteria has been demonstrated by several studies (Goodrich et al., 2014; Lim et al., 2017). Moreover, the functional study shows that a decreased number of Actinobacteria, especially the Bifidobacteria family (Duca et al., 2013), is associated with an enhancement of gut permeability, leading to the translocation of lipopolysaccharide into the serum. In addition, administration of *Bifidobacterium pseudocatenulatum* CECT 7765 along with a high-fat diet in mice can down-regulate the inflammation by reducing the production of inflammatory cytokines and chemokines (Cano et al., 2013), especially the TNF-α and IL-6. Of note, TNF-α and IL-6 contribute to reduced BMD (Zhao, 2018). Based on above evidence, it is safe to infer that the rs10920362 CT/TT genotype may increase the OP risk by reducing the abundance of Actinobacteria, Bifidobacteriaceae, and *Bifidobacterium*. It is also worth noting that the rs10920362 may be just a surrogate for some actually functional mQTLs in high LD with it. Overall, the result is of great encouragement and motivates future studies in this field to clarify the underlying biological mechanisms.

Our study is characterized by several strengths. First, it was first observed that two genetic variants as *LGR5* rs11178860 and *LGR6* rs10920362 were significantly associated with OP risk and/or BMDs and T-scores among Chinese elderly individuals. These findings provided new insights into R-Spondin/Wnt signaling pathway genes associated with the OP development. Second, to explore associations between gut microbiota composition and OP risk, our research took one step forward by analyzing more subjects. Third, we presented a systematic view of the associations among genetic variants, gut microbiota, and OP risk via integrative analysis of genome and microbiome data. We showed for the first time that the OP risk-related SNP (*LGR6* rs10920362) also associated with the abundance of disease-associated microbial taxa (Actinobacteria, Bifidobacteriaceae, and *Bifidobacterium*), providing potential directions for individualized OP prevention and treatment. Fourth, considering that the composition of gut microbiota is dynamic, complicated, and affected by immutable and modifiable factors (like dietary, prevalent diseases, and medications) (von Martels et al., 2017), we adjusted for potential confounders including sex, age, BMI, smoking and alcohol drinking. Last but not least, all participants were of Han ethnicity recruited from nearby communities in Wuhan, minimizing the heterogeneity of microbiota composition and host genetic architecture due to geographical factors (Mancabelli et al., 2017; Timpson et al., 2018).

However, several limitations should also be concerned. First, our study is of a cross-sectional design, failing to confirm a causal relationship. We did not conduct *in vitro* laboratory or *in vivo* animal studies to clarify underlying mechanisms for our findings. Moreover, large-scale prospective studies and functional experiments are warranted to elucidate the causality among the genetic variants, alterations of gut microbiota, and OP pathogenesis. As well, comprehensive researches of high quality are still needed to explore OP-related microbiome QTLs. Second, the participants recruited in this study were Chinese residents living in Wuhan, limiting the generalization of the study findings to other regions and other ethnic populations. Third, we adopted relative abundance indices to quantify the microbial composition (without absolute abundance measures), which was possibly inadequate enough to clarify disease-related microbiome alterations.

## Conclusion

Our data firstly supported that the *LGR5* rs11178860 and *LGR6* rs10920362 correlates with susceptibility of OP. Additionally, our comprehensive study of associations among R-spondin/Wnt signaling network gene polymorphisms, gut microbiota composition, and OP risk showed for the first time that the host genetic variant of rs10920362 in the *LGR6* gene may contribute to OP pathogenesis by reducing the relative abundance of Actinobacteria, Bifidobacteriaceae, and *Bifidobacterium*. The exploration of the interaction between host genes and gut microbiota provides new perspectives for the individualized and precise prevention and treatment of OP. To achieve better efficiency of OP prevention and treatment, the prospective microbiome-targeted therapeutics should take host genetic factors into consideration.

## Data Availability Statement

The data presented in the study are deposited in the National Center for Biotechnology Information (NCBI) Bioproject database with accession number PRJNA772039 (https://www.ncbi.nlm.nih.gov/bioproject/PRJNA772039).

## Ethics Statement

The studies involving human participants were reviewed and approved by the Ethics Committee of Tongji Medical College of Huazhong University of Science and Technology. The patients/participants provided their written informed consent to participate in this study.

## Author Contributions

QW, D-SD, CL, and YD had full access to the data in the study and take responsibility for the integrity of the data and the accuracy of the data analysis. QW, D-SD, and CL conceived the study. QW, D-SD, CL, and YD were responsible for conception of the study and drafted the manuscript. QW was responsible for design of the study. M-HW, S-SW, W-JS, H-LZ, YC, and R-YZ contributed to preparation and data analysis. QW, QH, D-SD, CL, and YD contributed to revision of the manuscript. All authors contributed to the interpretation of the data and critically reviewed the manuscript for publication. All authors read and approved the final manuscript.

## Conflict of Interest

The authors declare that the research was conducted in the absence of any commercial or financial relationships that could be construed as a potential conflict of interest.

## Publisher’s Note

All claims expressed in this article are solely those of the authors and do not necessarily represent those of their affiliated organizations, or those of the publisher, the editors and the reviewers. Any product that may be evaluated in this article, or claim that may be made by its manufacturer, is not guaranteed or endorsed by the publisher.

## Footnotes

1
https://github.com/FelixKrueger/TrimGalore

2
http://www.drive5.com/usearch/

## Supplementary Material

Supplementary Figure 1 Chromosome interactions and epigenetic signatures in bone related to the rs10920362 in 3DSNP database.

Supplementary Figure 2 Association between the rs10879301 and the splicing changes of LGR5 gene in the muscle skeletal tissue.

Supplementary Figure 3 Principal coordinate analysis (PCoA) based on weighted UniFrac distances comparing the bacterial community among the osteoporosis patients and controls groups.

Supplementary Figure 4 LEfSe indicating differences in the bacterial taxa at different levels (p, phylum; c, class; o, order; f, family; g, genus; and s, species). Only the taxes having a *P* < 0.05, and LDA value > 2 are shown in the figure.

Supplementary Table 1 Results of multiple linear regression analysis of the effects of the two top common variants on BMD measurements.

Supplementary Table 2 The HaploReg v4.1 predictions for the proxies of genetic variants rs11178860 and rs10920362.

Supplementary Table 3 Results of SNPinfo predictions for the proxies of genetic variants rs11178860 and rs10920362.

Supplementary Table 4 Results of the GTEx predictions for the genetic variant of rs10920362 with respect to gene expressions in various tissues.

Supplementary Table 5 Characteristics of included subjects for gut microbiota abundance analysis.

Supplementary Table 6 Bacterial compositions between the case and control groups at different levels.

Supplementary Table 7 Spearman correlation analysis between relative abundance of gut microbiota (at several taxonomic levels) and BMD measurements.

Supplementary Table 8 Ridge regression analysis of the effects of relative abundance of gut microbiota on BMD measurements.
