# Supplementary figures and images for: Integrative Analysis of LGR5/6 Gene Variants, Gut Microbiota Composition and Osteoporosis Risk in Elderly Population
Source: Front Microbiol. 2021 Nov 2;12:765008. doi: 10.3389/fmicb.2021.765008 (PMC8593465; doi:10.3389/fmicb.2021.765008)

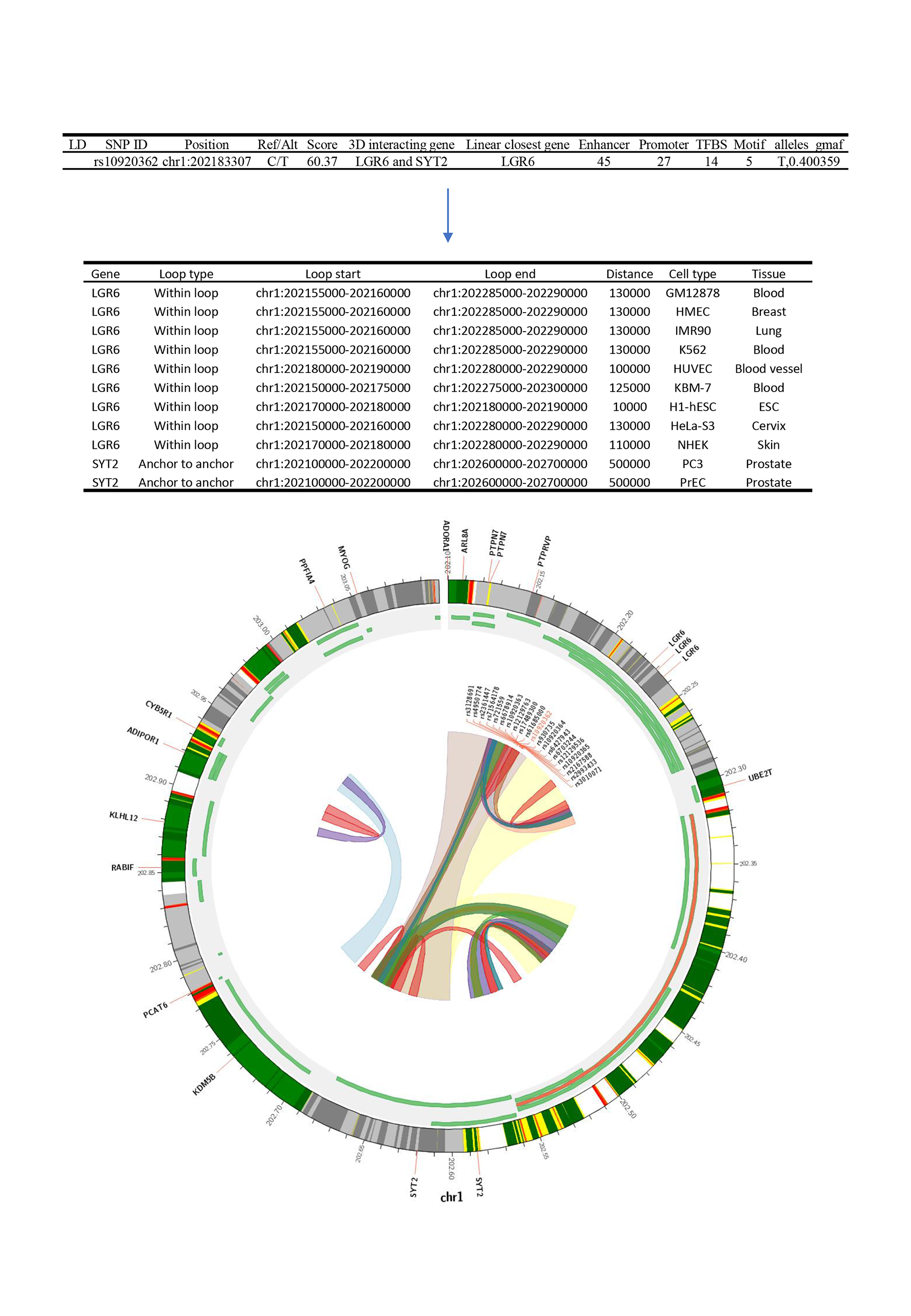

Supplement: Supplementary Figure 1 — Chromosome interactions and epigenetic signatures in bone related to the rs10920362 in 3DSNP database. [file Image_1.tif]

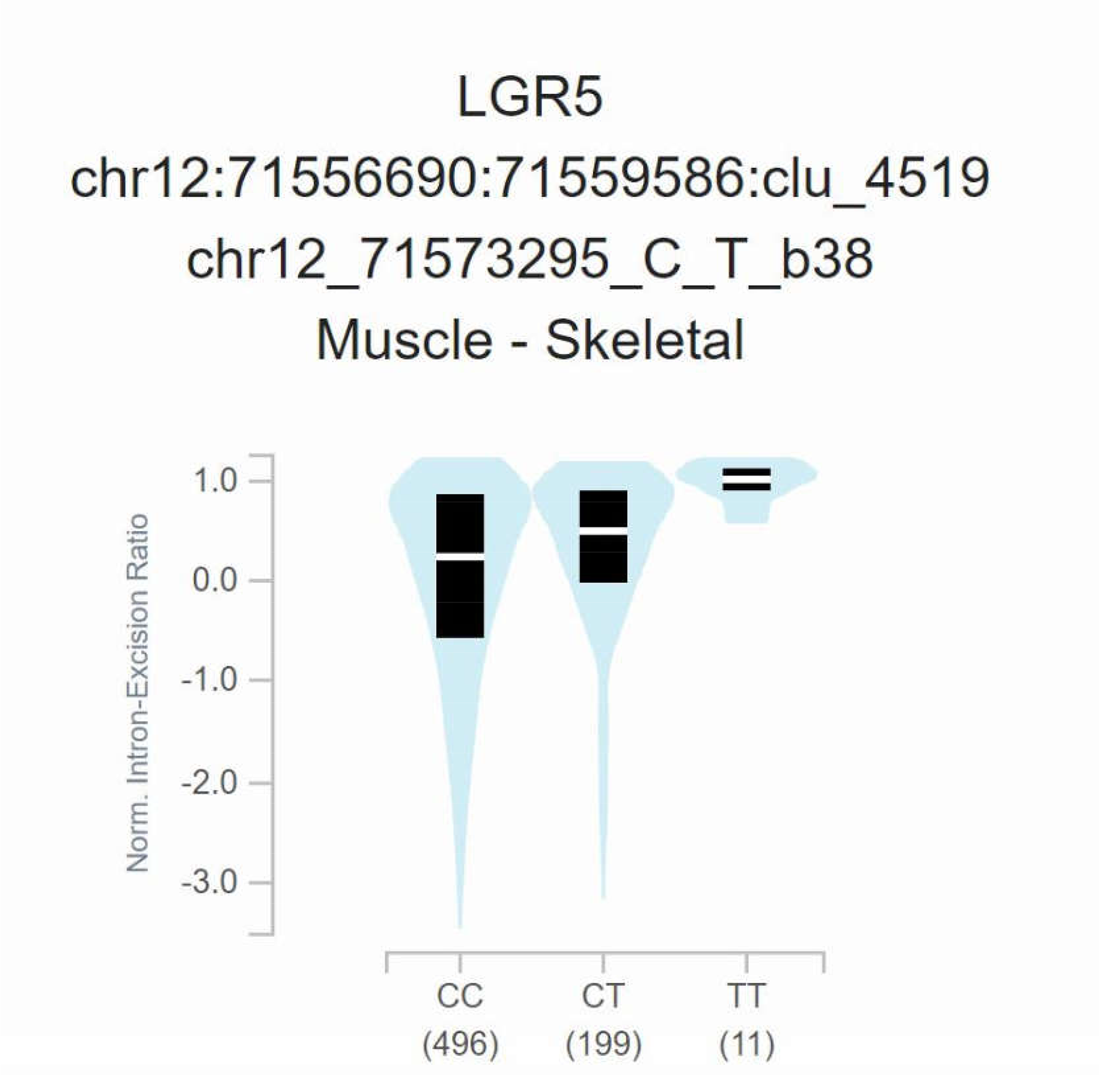

Supplement: Supplementary Figure 2 — Association between the rs10879301 and the splicing changes of LGR5 gene in the muscle skeletal tissue. [file Image_2.tif]

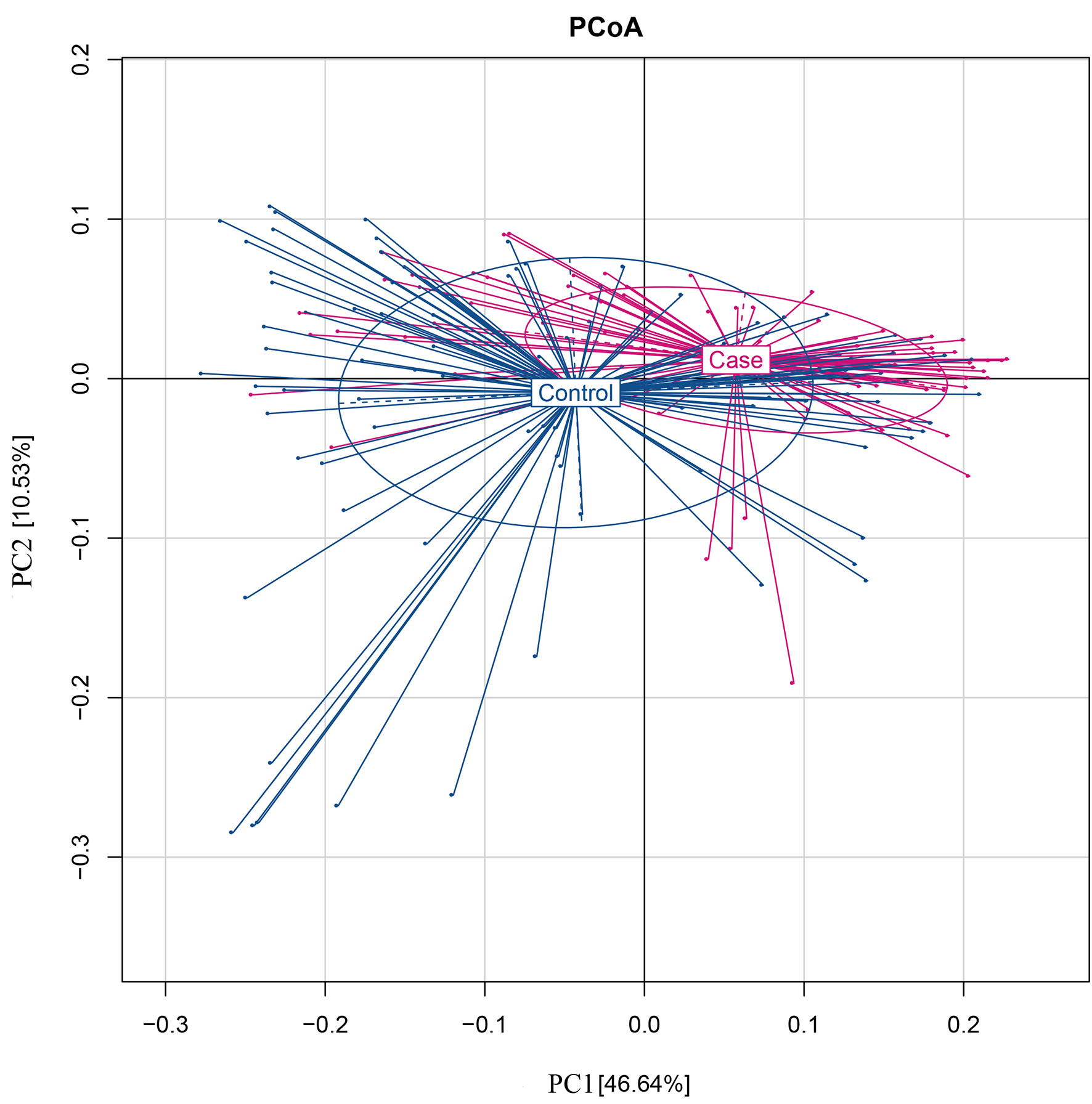

Supplement: Supplementary Figure 3 — Principal coordinate analysis (PCoA) based on weighted UniFrac distances comparing the bacterial community among the osteoporosis patients and controls groups. [file Image_3.tif]

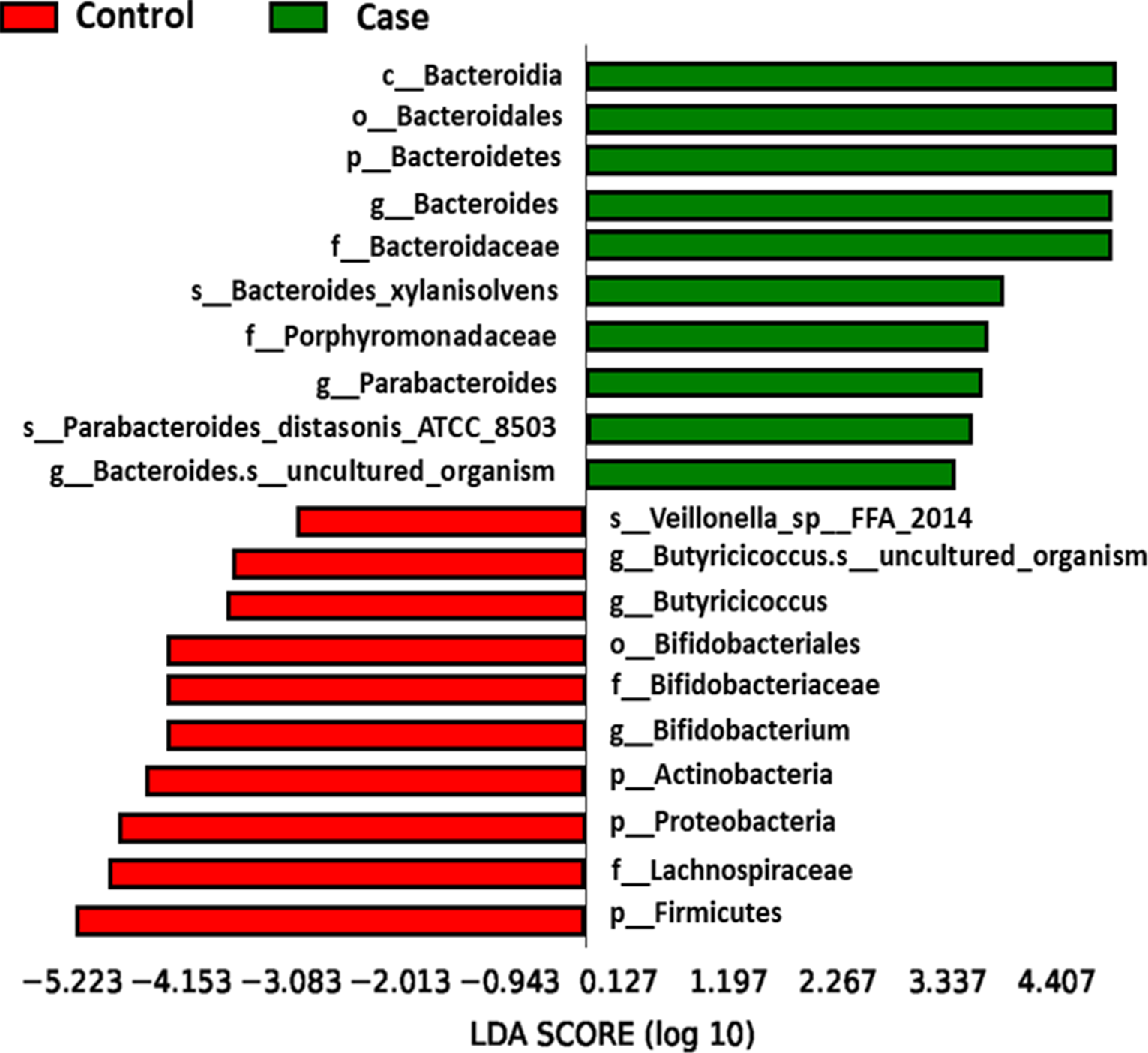

Supplement: Supplementary Figure 4 — LEfSe indicating differences in the bacterial taxa at different levels (p, phylum; c, class; o, order; f, family; g, genus; and s, species). Only the taxes having a P < 0.05, and LDA value > 2 are shown in the figure. [file Image_4.tif]
