# Supplementary material for: Integrative Analysis of LGR5/6 Gene Variants, Gut Microbiota Composition and Osteoporosis Risk in Elderly Population
Source: Front Microbiol. 2021 Nov 2;12:765008. doi: 10.3389/fmicb.2021.765008 (PMC8593465; doi:10.3389/fmicb.2021.765008)
Supplement: Supplementary Table 1 — Results of multiple linear regression analysis of the effects of the two top common variants on BMD measurements. [file Table_1.DOCX]

Table S1. Results of multiple linear regression analysis of the effects of the two top common variants on BMD measurements

| BMD measurement | rs10920362 | | rs11178860 | |
| --- | --- | --- | --- | --- |
|  | β (95% CI) | *P*-value/*P*-FDR | β (95% CI) | *P*-value/*P*-FDR |
| LS BMD (g/cm^2^) | -0.02 (-0.04, -0.01) | 0.006/0.031 | -0.02 (-0.03, -0.002) | 0.024/0.080 |
| LS T-score | -0.20 (-0.32, -0.07) | 0.003/0.013 | -0.14 (-0.25, -0.02) | 0.017/0.057 |
| LS Z-score | -0.20 (-0.32, -0.07) | 0.002/0.010 | -0.13 (-0.24, -0.02) | 0.023/0.060 |
| FN BMD (g/cm^2^) | -0.01 (-0.02, -0.001) | 0.038/0.188 | -0.01 (-0.02, -0.01) | 0.002/0.019 |
| FN T-score | -0.08 (-0.16, 0.01) | 0.089/0.337 | -0.10 (-0.17, -0.02) | 0.014/0.136 |
| FN Z-score | -0.09 (-0.17, -0.01) | 0.035/0.173 | -0.11 (-0.18, -0.03) | 0.004/0.039 |
| Total hip BMD (g/cm^2^) | -0.01 (-0.03, -0.003) | 0.010/0.051 | -0.01 (-0.02, 0.00) | 0.061/0.205 |
| Total hip T-score | -0.12 (-0.21, -0.03) | 0.008/0.039 | -0.08 (-0.16, -0.004) | 0.040/0.133 |
| Total hip Z-score | -0.12 (-0.21, -0.04) | 0.005/0.027 | -0.08 (-0.15, 0.001) | 0.054/0.183 |

Note：*P*-adjusted was Benjamini–Hochberg false discovery rate (FDR)-corrected. Adjusting covariates included sex, age, BMI, smoking and alcohol drinking.

LS, lumbar spine; FN, femoral neck; BMD bone mineral density
