# Supplementary material for: Integrative Analysis of LGR5/6 Gene Variants, Gut Microbiota Composition and Osteoporosis Risk in Elderly Population
Source: Front Microbiol. 2021 Nov 2;12:765008. doi: 10.3389/fmicb.2021.765008 (PMC8593465; doi:10.3389/fmicb.2021.765008)
Supplement: Supplementary Table 2 — The HaploReg v4.1 predictions for the proxies of genetic variants rs11178860 and rs10920362. [file Table_2.DOCX]

Table S2 The HaploReg v4.1 predictions for the proxies of genetic variants rs11178860 and rs10920362

| chr | LD(r²) | variant | Promoter histone marks | Enhancer histone marks | DNAse | Proteins bound | Motifs changed | GRASP QTL hits | Selected eQTL hits | GENCODE genes | dbSNP functional annotation |
| --- | --- | --- | --- | --- | --- | --- | --- | --- | --- | --- | --- |
| **12** | **1** | **rs11178860** | **BLD** | **BLD** |  |  | **NF-I, Nkx2** | **2 hits** | **1 hit** | ***LGR5*** | **intronic** |
| 12 | 0.97 | rs11178852 |  | BRST | BRST, SKIN |  | 5 altered motifs |  |  | *LGR5* | intronic |
| 12 | 0.97 | rs11178861 | BLD | BLD | BLD |  | Ets, NERF1a |  |  | *LGR5* | intronic |
| 12 | 0.97 | rs10879301 |  |  |  |  |  | 1 hit | 1 hit | *LGR5* | intronic |
| 12 | 0.97 | rs1880892 |  |  |  |  | Myf, Pbx-1 |  | 1 hit | *LGR5* | intronic |
| 12 | 0.95 | rs11178851 |  | BRST |  |  | XBP-1 |  |  | *LGR5* | intronic |
| 12 | 0.95 | rs11178853 |  | BRST | BRST |  |  |  | 1 hit | *LGR5* | intronic |
| 12 | 0.95 | rs11178854 |  |  |  |  | Pou2f2 |  |  | *LGR5* | intronic |
| 12 | 0.95 | rs10879298 |  |  |  |  | Foxp1, Pax-4, Pou2f2 |  | 1 hit | *LGR5* | intronic |
| 12 | 0.95 | rs7961238 |  |  |  |  | 7 altered motifs |  | 1 hit | *LGR5* | intronic |
| 12 | 0.95 | rs7976133 |  |  |  |  | HNF4, Ik-2 |  | 1 hit | *LGR5* | intronic |
| 12 | 0.95 | rs7976390 |  |  |  |  | Sox, TEF |  | 1 hit | *LGR5* | intronic |
| 12 | 0.95 | rs3816060 |  | BLD |  |  | PPAR, Pou2f2, Pou3f2 |  | 1 hit | *LGR5* | intronic |
| 12 | 0.95 | rs10506637 |  |  |  |  | SIX5 | 1 hit | 1 hit | *LGR5* | intronic |
| 12 | 0.95 | rs11178859 |  | BLD |  |  | ATF3, HNF4, SREBP |  |  | *LGR5* | intronic |
| 12 | 0.95 | rs7303838 |  | BLD |  |  | LUN-1, Pitx2, STAT |  |  | *LGR5* | intronic |
| 12 | 0.95 | rs7303989 |  | BLD |  |  | 13 altered motifs |  |  | *LGR5* | intronic |
| 12 | 0.95 | rs67774094 |  | BLD |  |  | 5 altered motifs |  |  | *LGR5* | intronic |
| 12 | 0.94 | rs2701084 |  | BRST |  |  | 4 altered motifs |  |  | *LGR5* | intronic |
| 12 | 0.94 | rs7964280 |  |  |  |  | 5 altered motifs |  | 1 hit | *LGR5* | intronic |
| 12 | 0.94 | rs10506636 |  |  |  |  | DMRT5 |  |  | *LGR5* | intronic |
| 12 | 0.94 | rs12812489 |  | BLD |  |  |  |  | 1 hit | *LGR5* | intronic |
| 12 | 0.94 | rs10879302 |  |  |  |  | 4 altered motifs |  |  | *LGR5* | intronic |
| 12 | 0.93 | rs10784927 |  |  |  |  | TATA |  |  | *LGR5* | intronic |
| 12 | 0.92 | rs145984633 |  |  |  |  | 9 altered motifs |  |  | *LGR5* | intronic |
| 12 | 0.92 | rs1148981 |  | BLD |  |  | 4 altered motifs |  |  | *LGR5* | intronic |
| 12 | 0.91 | rs12812625 |  | BLD |  |  | 5 altered motifs | 1 hit | 1 hit | *LGR5* | intronic |
| 12 | 0.88 | rs1280605 |  | BRN |  |  | ATF3 |  | 1 hit | *LGR5* | intronic |
| 12 | 0.88 | rs1280608 |  | ESC, IPSC, BRN | IPSC, BRN |  | Nanog, YY1 |  | 1 hit | *LGR5* | intronic |
| 12 | 0.88 | rs1280611 |  | ESC, IPSC, BRN |  |  | MAZ, VDR |  | 1 hit | *LGR5* | intronic |
| 12 | 0.87 | rs7296236 |  |  | BLD |  | 4 altered motifs |  |  | *LGR5* | intronic |
| 12 | 0.81 | rs111375633 |  | BLD |  |  | 5 altered motifs |  |  | *LGR5* | intronic |
| **1** | **1** | **rs10920362** |  |  | **9 tissues** | **POL2** | **4 altered motifs** | **3 hits** | **3 hits** | ***LGR6*** | **missense** |
| 1 | 0.96 | rs930735 |  | 6 tissues | PLCNT, GI, LNG | CEBPB | CHOP: CEBPalpha, Ik-2 |  | 5 hits | *LGR6* | intronic |
| 1 | 0.95 | rs10920363 |  |  | PLCNT |  | 8 altered motifs |  | 3 hits | *LGR6* | intronic |
| 1 | 0.96 | rs10920364 |  |  | PLCNT |  | GR |  | 5 hits | *LGR6* | intronic |
| 1 | 0.95 | rs721559 |  | BRST, BRN, VAS |  |  | Gm397, HDAC2, NRSF |  | 4 hits | *LGR6* | intronic |
| 1 | 0.86 | rs2361446 |  | BRST, BRN, VAS |  |  | Zbtb3 |  | 1 hit | *LGR6* | intronic |
| 1 | 0.86 | rs2924105 |  | BRST, BRN |  |  | 6 altered motifs | 1 hit | 2 hits | *LGR6* | intronic |
| 1 | 0.86 | rs2361447 |  | BRST, THYM |  |  | Myc, RREB-1 |  | 2 hits | *LGR6* | intronic |
| 1 | 0.84 | rs3010073 |  | BRST, THYM |  | GATA3 | CCNT2, GATA |  | 2 hits | *LGR6* | intronic |
| 1 | 0.83 | rs2993433 | THYM | 12 tissues |  |  | Pou5f1, Zfp410 |  | 2 hits | *LGR6* | intronic |
| 1 | 0.83 | rs3010071 | THYM | 12 tissues | THYM |  | 7 altered motifs | 1 hit | 2 hits | *LGR6* | intronic |
| 1 | 0.84 | rs6681437 |  |  |  |  | CDP, Myc, SREBP | 1 hit | 4 hits | *snoU13* | intronic |
