# Supplementary material for: Integrative Analysis of LGR5/6 Gene Variants, Gut Microbiota Composition and Osteoporosis Risk in Elderly Population
Source: Front Microbiol. 2021 Nov 2;12:765008. doi: 10.3389/fmicb.2021.765008 (PMC8593465; doi:10.3389/fmicb.2021.765008)
Supplement: Supplementary Table 3 — Results of SNPinfo predictions for the proxies of genetic variants rs11178860 and rs10920362. [file Table_3.DOCX]

Table S3 Results of SNPinfo predictions for the proxies of genetic variants rs11178860 and rs10920362.

| rs | Chr | Position | Allele | LDsnp | Pop/LD | TFBS | Splicing (ESE or ESS) | nsSNP | Polyphen | RegPotential | Conservation | Nearby Gene |
| --- | --- | --- | --- | --- | --- | --- | --- | --- | --- | --- | --- | --- |
| **rs10920362** | **1** | **200449931** | **C/T** | **rs10920362** | **1** | **--** | **Y** | **Y** | **Possibly damaging** | **1** | **0** | ***LGR6*** |
| rs10920364 | 1 | 200452801 | C/T | rs10920362 | CHB/1 | -- | -- | -- | -- | 0.151669 | 0 | *LGR6* |
| rs12033460 | 1 | 200446837 | A/G | rs10920362 | CHB/0.806 | -- | -- | -- | -- | 0 | 0.006 | *LGR6* |
| rs12129536 | 1 | 200455961 | C/T | rs10920362 | CHB/0.857 | -- | -- | -- | -- | NA | 0 | *LGR6* |
| rs2361447 | 1 | 200456387 | C/T | rs10920362 | CHB/0.862 | -- | -- | -- | -- | 0 | 0.001 | *LGR6* |
| rs2924105 | 1 | 200455264 | C/T | rs10920362 | CHB/0.886 | -- | -- | -- | -- | 0 | 0.001 | *LGR6* |
| rs2993433 | 1 | 200457365 | C/T | rs10920362 | CHB/0.819 | -- | -- | -- | -- | 0 | 0 | *LGR6* |
| rs3010071 | 1 | 200457408 | T/A | rs10920362 | CHB/0.823 | -- | -- | -- | -- | 0 | 0 | *LGR6* |
| rs3010073 | 1 | 200456589 | G/A | rs10920362 | CHB/0.886 | -- | -- | -- | -- | 0 | 0 | *LGR6* |
| rs6681437 | 1 | 200467435 | A/G | rs10920362 | CHB/0.839 | -- | Y | -- | -- | NA | 0 | *LGR6* |
| rs721559 | 1 | 200454742 | T/C | rs10920362 | CHB/1 | -- | -- | -- | -- | 0 | 0 | *LGR6* |
| rs930735 | 1 | 200452602 | A/G | rs10920362 | CHB/1 | -- | -- | -- | -- | 0.069686 | 0 | *LGR6* |
| rs10506636 | 12 | 70251357 | A/G | rs11178860 | CHB/1 | -- | -- | -- | -- | 0 | 0.509 | *LGR5* |
| rs10506637 | 12 | 70252083 | C/T | rs11178860 | CHB/1 | -- | -- | -- | -- | 0 | 0 | *LGR5* |
| rs10784927 | 12 | 70249242 | A/G | rs11178860 | CHB/1 | -- | -- | -- | -- | NA | 0 | *LGR5* |
| rs10879301 | 12 | 70253342 | C/T | rs11178860 | CHB/1 | -- | -- | -- | -- | 0 | 0 | *LGR5* |
| rs11178853 | 12 | 70250253 | A/G | rs11178860 | CHB/1 | -- | -- | -- | -- | NA | 0 | *LGR5* |
| **rs11178860** | **12** | **70253103** | **A/G** | **rs11178860** | **1** | **--** | **--** | **--** | **--** | **0.012029** | **0.747** | ***LGR5*** |
| rs1280605 | 12 | 70242584 | C/T | rs11178860 | CHB/0.976 | -- | -- | -- | -- | NA | 0 | *LGR5* |
| rs1280608 | 12 | 70243503 | G/T | rs11178860 | CHB/1 | -- | -- | -- | -- | 0 | 0 | *LGR5* |
| rs12812489 | 12 | 70252618 | A/C | rs11178860 | CHB/1 | -- | -- | -- | -- | 0 | 0 | *LGR5* |
| rs12812625 | 12 | 70252531 | A/G | rs11178860 | CHB/1 | -- | -- | -- | -- | 0 | 0.006 | *LGR5* |
| rs17110150 | 12 | 70349549 | A/G | rs11178860 | CHB/1 | -- | -- | -- | -- | 0 | 0.077 | *THAP2* |
| rs1880892 | 12 | 70254077 | C/T | rs11178860 | CHB/1 | -- | -- | -- | -- | NA | 0 | *LGR5* |
| rs2304269 | 12 | 70366539 | C/T | rs11178860 | CHB/0.814 | Y | -- | -- | -- | 0 | 0 | *TMEM19* |
| rs7305505 | 12 | 70293351 | A/G | rs11178860 | CHB/0.927 | -- | -- | -- | -- | 0.019858 | 0.001 | *CCDC131* |
| rs7961238 | 12 | 70250651 | A/G | rs11178860 | CHB/1 | -- | -- | -- | -- | 0.050884 | 0 | *LGR5* |
| rs7964280 | 12 | 70250980 | C/T | rs11178860 | CHB/1 | -- | -- | -- | -- | 0.100174 | 0.997 | *LGR5* |
| rs7967428 | 12 | 70375307 | A/G | rs11178860 | CHB/0.837 | -- | -- | -- | -- | 0 | 0 | *TMEM19* |
| rs7976133 | 12 | 70250735 | C/T | rs11178860 | CHB/1 | -- | -- | -- | -- | 0.000253 | 0.289 | *LGR5* |
| rs7976390 | 12 | 70250933 | G/T | rs11178860 | CHB/1 | -- | -- | -- | -- | 0.11785 | 0 | *LGR5* |
