# Supplementary material for: Integrative Analysis of LGR5/6 Gene Variants, Gut Microbiota Composition and Osteoporosis Risk in Elderly Population
Source: Front Microbiol. 2021 Nov 2;12:765008. doi: 10.3389/fmicb.2021.765008 (PMC8593465; doi:10.3389/fmicb.2021.765008)
Supplement: Supplementary Table 4 — Results of the GTEx predictions for the genetic variant of rs10920362 with respect to gene expressions in various tissues. [file Table_4.DOCX]

| Gencode Id | Gene Symbol | Variant Id | SNP Id | *P*-Value | Tissue |
| --- | --- | --- | --- | --- | --- |
| ENSG00000133067.17 | *LGR6* | chr1_202214180_C_T_b38 | rs10920362 | 7.20 × 10^-39^ | Thyroid |
| ENSG00000133067.17 | *LGR6* | chr1_202214180_C_T_b38 | rs10920362 | 1.10 × 10^-11^ | Brain - Hypothalamus |
| ENSG00000133067.17 | *LGR6* | chr1_202214180_C_T_b38 | rs10920362 | 2.10 × 10^-9^ | Minor Salivary Gland |
| ENSG00000133067.17 | *LGR6* | chr1_202214180_C_T_b38 | rs10920362 | 1.00 × 10^-8^ | Artery - Tibial |
| ENSG00000133067.17 | *LGR6* | chr1_202214180_C_T_b38 | rs10920362 | 3.00 × 10^-7^ | Brain - Substantia nigra |
| ENSG00000133067.17 | *LGR6* | chr1_202214180_C_T_b38 | rs10920362 | 4.20 × 10^-7^ | Artery - Aorta |
| ENSG00000133067.17 | *LGR6* | chr1_202214180_C_T_b38 | rs10920362 | 4.30 × 10^-5^ | Brain - Cortex |

Table S4 Results of the GTEx predictions for the genetic variant of rs10920362 with respect to gene expressions in various tissues.
