# Supplementary material for: Integrative Analysis of LGR5/6 Gene Variants, Gut Microbiota Composition and Osteoporosis Risk in Elderly Population
Source: Front Microbiol. 2021 Nov 2;12:765008. doi: 10.3389/fmicb.2021.765008 (PMC8593465; doi:10.3389/fmicb.2021.765008)
Supplement: Supplementary Table 5 — Characteristics of included subjects for gut microbiota abundance analysis. [file Table_5.DOCX]

Table S5. Characteristics of included subjects for gut microbiota abundance analysis

| Variables | N | Cases (n=77) | Controls (n=103) | *P*-value |
| --- | --- | --- | --- | --- |
| Age (years), mean ± SD | 180 | 67.0±6.7 | 66.1±7.2 | 0.301 |
| Female, n (%) | 180 | 56(72.7%) | 47(45.6%) | 2.78×10^-4^ |
| BMI (kg/m^2^), mean ± SD | 180 | 23.07±3.37 | 24.44±4.41 | 0.006 |
| smoking (%) | 180 | 27(35.1%) | 21(20.4%) | 0.028 |
| drinking (%) | 180 | 25(32.5%) | 23(22.3%) | 0.128 |
| Fracture (%) | 178 | 28(36.8%) | 26(25.5%) | 0.103 |
| LS BMD, mean ± SD (g/cm^2^) | 180 | 0.89±0.12 | 1.13±0.16 | 3.06×10^-23^ |
| LS T-score | 180 | -2.51±0.97 | -0.45±1.26 | 9.14×10^-26^ |
| LS Z-score | 180 | -0.83±0.93 | 0.78±1.23 | 9.34×10^-18^ |
| FN BMD, mean ± SD (g/cm^2^) | 180 | 0.71±0.09 | 0.87±0.11 | 1.78×10^-19^ |
| FN T-score | 180 | -2.40±1.00 | -1.30±1.00 | 6.97×10^-16^ |
| FN Z-score | 180 | -0.70±0.90 | 0.20±1.10 | 1.72×10^-12^ |
| Hip BMD, mean ± SD (g/cm^2^) | 180 | 0.78±0.10 | 0.95±0.12 | 4.17×10^-20^ |
| Hip T-score | 179 | -2.00±1.05 | -0.70±1.30 | 2.74×10^-18^ |
| Hip Z-score | 180 | -0.68±0.87 | 0.39±0.86 | 4.88×10^-14^ |

Note: SD, standard deviation; LS, lumbar spine; FN, femoral neck; BMD bone mineral density.
