# Supplementary material for: Integrative Analysis of LGR5/6 Gene Variants, Gut Microbiota Composition and Osteoporosis Risk in Elderly Population
Source: Front Microbiol. 2021 Nov 2;12:765008. doi: 10.3389/fmicb.2021.765008 (PMC8593465; doi:10.3389/fmicb.2021.765008)
Supplement: Supplementary Table 6 — Bacterial compositions between the case and control groups at different levels. [file Table_6.DOCX]

Table S6. Bacterial compositions between the case and control groups at different levels.

| Group | Phylum | Class | Order | Family | Genus | Species | OTU |
| --- | --- | --- | --- | --- | --- | --- | --- |
| Cases | 21 | 41 | 54 | 112 | 267 | 333 | 1288 |
| Controls | 25 | 47 | 64 | 124 | 303 | 396 | 1556 |
| Total | 25 | 47 | 64 | 126 | 318 | 426 | 1699 |

Note: OTU, operational taxonomic unit. Values in the “total” row indicate a summarized number of phylum, class, order, family, genus, species, and OUT respectively, across all fecal samples in this study.
