# Supplementary material for: Integrative Analysis of LGR5/6 Gene Variants, Gut Microbiota Composition and Osteoporosis Risk in Elderly Population
Source: Front Microbiol. 2021 Nov 2;12:765008. doi: 10.3389/fmicb.2021.765008 (PMC8593465; doi:10.3389/fmicb.2021.765008)
Supplement: Supplementary Table 7 — Spearman correlation analysis between relative abundance of gut microbiota (at several taxonomic levels) and BMD measurements. [file Table_7.DOCX]

#### Table S7. Spearman correlation analysis between relative abundance of gut microbiota (at several taxonomic levels) and BMD measurements

| Taxonomic level | LS BMD | LS T-score | LS Z-score | FN BMD | FN T-score | FN Z-score | Hip BMD | Hip T-score | Hip Z-score |
| --- | --- | --- | --- | --- | --- | --- | --- | --- | --- |
| p-Firmicutes | 0.200** | 0.217** | 0.173* | 0.143 | 0.160* | 0.141 | 0.132 | 0.145 | 0.084 |
| p-Bacteroidetes | -0.276** | -0.289** | -0.239** | -0.230** | -0.217** | -0.206** | -0.225** | -0.218** | -0.169* |
| p-Proteobacteria | 0.196** | 0.200** | 0.184* | 0.156* | 0.110 | 0.101 | 0.189* | 0.198** | 0.168* |
| p-Actinobacteria | 0.199** | 0.201** | 0.184* | 0.187* | 0.194** | 0.167* | 0.172* | 0.151* | 0.135 |
| f-Bacteroidaceae | -0.300** | -0.305** | -0.232** | -0.278** | -0.258** | -0.226** | -0.253** | -0.233** | -0.182* |
| f-Lachnospiraceae | 0.159* | 0.180* | 0.163* | 0.076 | 0.102 | 0.094 | 0.115 | 0.145 | 0.104 |
| f-Ruminococcaceae | 0.197** | 0.177* | 0.119 | 0.168* | 0.155* | 0.123 | 0.165* | 0.136 | 0.078 |
| f-Enterobacteriaceae | 0.132 | 0.150* | 0.146 | 0.047 | 0.003 | 0.030 | 0.054 | 0.063 | 0.036 |
| f-Bifidobacteriaceae | 0.224** | 0.228** | 0.208** | 0.196** | 0.200** | 0.175* | 0.193** | 0.177* | 0.165* |
| f-Lactobacillaceae | 0.240** | 0.257** | 0.208** | 0.163* | 0.156* | 0.115 | 0.145 | 0.128 | 0.078 |
| g-Bacteroides | -0.300** | -0.305** | -0.232** | -0.278** | -0.258** | -0.226** | -0.253** | -0.233** | -0.182* |
| g-Lachnospiracea  _incertae_sedis | 0.190* | 0.194** | 0.146 | 0.101 | 0.115 | 0.069 | 0.166* | 0.167* | 0.129 |
| g-Bifidobacterium | 0.224** | 0.228** | 0.209** | 0.196** | 0.200** | 0.175* | 0.194** | 0.178* | 0.166* |
| g-Parabacteroides | -0.139 | -0.156* | -0.110 | -0.096 | -0.089 | -0.055 | -0.093 | -0.105 | -0.053 |
| g-Ruminococcus | 0.140 | 0.156* | 0.133 | 0.085 | 0.104 | 0.055 | 0.119 | 0.113 | 0.071 |
| g-Clostridium_XlVa | -0.132 | -0.116 | -0.083 | -0.250** | -0.215** | -0.215** | -0.163* | -0.113 | -0.121 |
| g-Lactobacillus | 0.240** | 0.257** | 0.208** | 0.163* | 0.156* | 0.115 | 0.145 | 0.128 | 0.078 |
| g-Gemmiger | 0.275** | 0.261** | 0.197** | 0.236** | 0.261** | 0.202** | 0.234** | 0.225** | 0.173* |
| g-Dialister | 0.162* | 0.150* | 0.121 | 0.125 | 0.129 | 0.108 | 0.139 | 0.148* | 0.108 |

*Note*: Only the phylum, family, and genera with relative abundance greater than 0.1% were included in this analysis. **P*<0.05; ***P*<0.01.

LS, lumbar spine; FN, femoral neck; p, phylum; c, class; o, order; f, family; g, genus.
