# Supplementary material for: Integrative Analysis of LGR5/6 Gene Variants, Gut Microbiota Composition and Osteoporosis Risk in Elderly Population
Source: Front Microbiol. 2021 Nov 2;12:765008. doi: 10.3389/fmicb.2021.765008 (PMC8593465; doi:10.3389/fmicb.2021.765008)
Supplement: Supplementary Table 8 — Ridge regression analysis of the effects of relative abundance of gut microbiota on BMD measurements. [file Table_8.DOCX]

Table S8. Ridge regression analysis of the effects of relative abundance of gut microbiota on BMD measurements

| Taxonomic level | LS BMD | LS T-score | LS Z-score | FN BMD | FN T-score | FN Z-score | Hip BMD | Hip T-score | Hip Z-score |
| --- | --- | --- | --- | --- | --- | --- | --- | --- | --- |
| p-Firmicutes | 0.021 | 0.349 | 0.291 | -0.012 | 1.493 | 0.120 | 0.013 | 3.908* | 0.069 |
| p-Bacteroidetes | -0.054 | -0.567* | -0.457* | -0.046 | -0.624 | -0.350 | -0.038 | -2.576 | -0.240 |
| p-Proteobacteria | 0.157 | 1.296 | 1.211 | 0.066 | -1.152 | 0.298 | 0.118 | 2.333 | 1.022 |
| p-Actinobacteria | 0.072 | 0.384 | 0.703 | 0.093 | -1.832 | 0.439 | 0.079 | -3.588 | 0.348 |
| f-Bacteroidaceae | -0.126 | -1.020* | -0.950* | -0.114* | -2.052 | -0.852* | -0.112 | 0.567 | -0.709* |
| f-Lachnospiraceae | 0.035 | 0.781 | 0.790 | -0.055 | 0.542 | -0.100 | -0.008 | -6.551* | -0.082 |
| f-Ruminococcaceae | 0.045 | 0.321 | 0.242 | -0.010 | 5.730 | 0.683 | -0.003 | -0.192 | 0.139 |
| f-Enterobacteriaceae | 0.147 | 1.242 | 1.284 | 0.020 | -0.473 | 0.232 | 0.060 | 4.623 | 0.805 |
| f-Porphyromonadaceae | 0.093 | 1.174 | 1.667 | -0.061 | 2.039 | 0.368 | 0.026 | -2.006 | 0.654 |
| f-Bifidobacteriaceae | 0.109 | 0.847 | 1.055 | 0.130 | -2.406 | 0.700 | 0.123 | -4.580 | 0.400 |
| f-Lactobacillaceae | 0.124 | 1.808 | 1.537 | 0.072 | 1.489 | 0.785 | -0.055 | 2.803 | -0.389 |
| g-Bacteroides | -0.084 | -0.698 | -0.677 | -0.098* | -1.560 | -0.889 | -0.080 | -8.450** | -0.721 |
| g-Lachnospiracea_  incertae_sedis | -0.009 | 0.322 | 0.119 | -0.047 | 9.843 | 0.732 | 0.073 | -13.08 | -0.012 |
| g-Bifidobacterium | 0.146 | 1.105 | 1.189 | 0.131 | -3.046 | 0.519 | 0.119 | -7.720 | 0.135 |
| g-Parabacteroides | -0.010 | 0.425 | 0.886 | 0.033 | 2.304 | 0.740 | 0.016 | -12.56 | 0.512 |
| g-Ruminococcus | -0.175 | -0.196 | 0.404 | -0.371 | -1.766 | -2.089 | -0.387 | -20.29 | -2.636 |
| g-Clostridium_XlVa | -0.226 | -1.781 | -1.598 | -0.321 | -14.66 | -4.745 | -0.479 | -16.47 | -3.519 |
| g-Lactobacillus | 0.096 | 1.592 | 1.245 | 0.078 | 2.275 | 0.766 | -0.036 | -6.106 | -0.527 |
| g-Gemmiger | 0.759 | 5.381 | 4.965 | 0.147 | 0.999 | -0.560 | 0.375 | 31.35 | 3.748 |
| g-Dialister | 0.449 | 3.319 | 2.644 | 0.245 | 12.82 | 2.422 | 0.267 | -12.64 | 1.222 |

Note: The ridge regression model was adjusted for age, sex, cigarette smoking, and body mass index. Only the phylum, family, and genus with relative abundance greater than 0.1% were included in this analysis. **P*<0.05; ***P*<0.01.

LS, lumbar spine; FN, femoral neck; p, phylum; c, class; o, order; f, family; g, genus.
